# Recommendations for Clinicians, Technologists, and Healthcare Organizations on the Use of Generative Artificial Intelligence in Medicine: A Position Statement from the Society of General Internal Medicine

**DOI:** 10.1007/s11606-024-09102-0

**Published:** 2024-11-12

**Authors:** Byron Crowe, Shreya Shah, Derek Teng, Stephen P. Ma, Matthew DeCamp, Eric I. Rosenberg, Jorge A. Rodriguez, Benjamin X. Collins, Kathryn Huber, Kyle Karches, Shana Zucker, Eun Ji Kim, Lisa Rotenstein, Adam Rodman, Danielle Jones, Ilana B. Richman, Tracey L. Henry, Diane Somlo, Samantha I. Pitts, Jonathan H. Chen, Rebecca G. Mishuris

**Affiliations:** 1 Division of General Internal Medicine, Beth Israel Deaconess Medical Center, Boston, MA USA; 2 Harvard Medical School, Boston, MA USA; 3 Department of Medicine, Stanford University, Palo Alto, CA USA; 4 Division of Primary Care and Population Health, Stanford Healthcare AI Applied Research Team, Stanford University School of Medicine, Palo Alto, CA USA; 5 Department of Medicine, University of Colorado, Aurora, CO USA; 6 Division of General Internal Medicine, Department of Medicine, University of Florida College of Medicine, Gainesville, FL USA; 7 Division of General Internal Medicine, Brigham and Women’s Hospital, Boston, MA USA; 8 Division of General Internal Medicine and Public Health, Vanderbilt University Medical Center, Nashville, TN USA; 9 Department of Internal Medicine, Kaiser Permanente, Denver, CO, School of Medicine, University of Colorado, Aurora, CO USA; 10 Department of Internal Medicine, Saint Louis University, Saint Louis, MO USA; 11 Department of Internal Medicine, University of Miami Miller School of Medicine, Jackson Memorial Hospital, Miami, FL USA; 12 Northwell Health, New Hyde Park, NY USA; 13 Divisions of General Internal Medicine and Clinical Informatics, Department of Medicine, University of California at San Francisco, San Francisco, CA USA; 14 Division of General Internal Medicine, Emory University School of Medicine, Atlanta, GA USA; 15 Section of General Internal Medicine, Yale School of Medicine, New Haven, CT USA; 16 Department of Medicine, Massachusetts General Hospital, Boston, MA USA; 17 Division of General Internal Medicine, Johns Hopkins University School of Medicine, Baltimore, MD USA; 18 Stanford Center for Biomedical Informatics Research, Stanford, CA USA; 19 Division of Hospital Medicine, Stanford, CA USA; 20 Clinical Excellence Research Center, Stanford, CA USA; 21 Digital, Mass General Brigham, Somerville, MA USA; 22 Department of Biomedical Informatics, Vanderbilt University, Nashville, TN USA

**Keywords:** clinical practice, artificial intelligence, healthcare technology

## Abstract

Generative artificial intelligence (generative AI) is a new technology with potentially broad applications across important domains of healthcare, but serious questions remain about how to balance the promise of generative AI against unintended consequences from adoption of these tools. In this position statement, we provide recommendations on behalf of the Society of General Internal Medicine on how clinicians, technologists, and healthcare organizations can approach the use of these tools. We focus on three major domains of medical practice where clinicians and technology experts believe generative AI will have substantial immediate and long-term impacts: clinical decision-making, health systems optimization, and the patient-physician relationship. Additionally, we highlight our most important generative AI ethics and equity considerations for these stakeholders. For clinicians, we recommend approaching generative AI similarly to other important biomedical advancements, critically appraising its evidence and utility and incorporating it thoughtfully into practice. For technologists developing generative AI for healthcare applications, we recommend a major frameshift in thinking away from the expectation that clinicians will “supervise” generative AI. Rather, these organizations and individuals should hold themselves and their technologies to the same set of high standards expected of the clinical workforce and strive to design high-performing, well-studied tools that improve care and foster the therapeutic relationship, not simply those that improve efficiency or market share. We further recommend deep and ongoing partnerships with clinicians and patients as necessary collaborators in this work. And for healthcare organizations, we recommend pursuing a combination of both incremental and transformative change with generative AI, directing resources toward both endeavors, and avoiding the urge to rapidly displace the human clinical workforce with generative AI. We affirm that the practice of medicine remains a fundamentally human endeavor which should be enhanced by technology, not displaced by it.

**Supplementary Information:**

The online version contains supplementary material available at 10.1007/s11606-024-09102-0.

## INTRODUCTION

Generative artificial intelligence (generative AI) recently emerged as a major technology breakthrough. Although “AI” is a broad term, generative AI encompasses a specific set of new tools with advanced capabilities in interpreting and manipulating natural language. Conceptually, generative AI has been compared to other world-changing technologies like the modern Internet and smartphones, fostering robust discussion on societal implications. Although others have written extensively on *how* generative AI works,^1^ we focus primarily on *why* this technology is different and ways its application may shape internal medicine and healthcare more broadly.

Briefly, generative AI capabilities are based on a new class of advanced data models “trained” on data such as texts, images, and audio at a massive scale, on the order of billions to trillions of associations spanning the breadth of existing human knowledge.^2^ These models are then refined through various technical methods to satisfy human preferences and accomplish specific tasks. By leveraging such a large corpus of information and drawing patterns between the data, generative AI can *generate* new content in response to diverse inputs. We provide several illustrative examples of generative AI output (Supplementary Appendix 1). For text-based responses like the examples provided, generative AI literally predicts the most likely next word in a sentence — but this is an oversimplification and does not do justice to their extensive capabilities. We suggest readers interact firsthand with these widely available tools to appreciate their power and limitations.

The most important concept to internalize for physicians is that generative AI tools can interpret and create content from diverse inputs while exhibiting the ability to reason^3,4^ — domains historically reserved largely for human experts. Generative AI can now complete tasks which previously required substantial human effort and skill such as answering complex questions, summarizing large documents, interpreting and creating images and audio, and much more. In medicine, generative AI is uniquely positioned to address many challenges facing clinicians and patients.^5^ However, this sense of optimism must be weighed against unknown impacts of generative AI on healthcare quality, safety, equity, and ethics.^6,7^ Important questions remain about how this technology will affect care delivery.

In this position statement, we make recommendations on behalf of the Society of General Internal Medicine (SGIM) on the use of generative AI in medicine. We convened a group of clinical, health systems, and technology experts within SGIM to explore three domains of clinical practice where application of generative AI may substantially impact care delivery: clinical decision-making, health systems optimization, and the patient-physician relationship. These categories were selected by expert consensus within the writing group and are consistent with domains identified by the physician community as areas of enthusiasm and concern.^8^ Additionally, we provide an overview of our most pressing ethical and equity concerns surrounding generative AI implementation. In each section, we review generative AI’s potential, highlight key challenges to overcome, and provide actionable recommendations to three groups: clinicians using these tools in frontline patient care, technologists developing these tools for healthcare applications, and healthcare organizations making decisions on adoption of generative AI technology. Each of these categories is composed of many stakeholders but includes individual practitioners, technology companies, health plans, purchasers of healthcare services, and provider organizations like health systems and clinics. We also use the term “industry” throughout when specifically referring to organizations selling generative AI tools for financial gain. While much has already been written about the potential of generative AI in healthcare,^8^ our views represent the unique perspective of internal medicine physicians, the largest physician specialty in the United States.^9^ Although we anticipate these recommendations will evolve as this technology advances, they are grounded in well-established principles for achieving a high-performing healthcare system including safety, timeliness, effectiveness, efficiency, equity, and patient-centeredness.^10^

These recommendations were collaboratively developed by the SGIM committees on Clinical Practice, Health Policy, Ethics, Health Equity, and Research. They were approved by the SGIM Council on April 5, 2024.

## ENHANCING CLINICAL DECISION-MAKING WITH GENERATIVE AI

Clinical decision-making is a complex cognitive process that is foundational to the practice of medicine. At its most basic level, it may be conceptualized as collecting, organizing, and interpreting information to make a diagnosis and select appropriate treatment. When done well, it also requires application of expert knowledge alongside years of hard-earned experience, judgment in the face of uncertainty, and a deep appreciation of the values, goals, and circumstances of our patients.

Generative AI may support clinical decision-making through analysis of multimodal clinical data and generation of personalized insights into diagnostic and treatment options which reflect the most current medical knowledge. Such tools have already shown impressive performance in diagnostic reasoning, demonstrating the ability to surface correct diagnoses in complex diagnostic challenges^11^ and compare favorably to human performance in simulated medical cases.^12,13^ Studies of real-world implementations of analytic AI have demonstrated strong physician agreement with AI-generated differential diagnoses in internal medicine settings, though important areas of discordance were identified.^14^ Better diagnostic supports would be a welcome contribution given that diagnostic harm affects nearly 5% of encounters for outpatients^15^ and 0.7% of encounters for inpatients.^16^

In addition to diagnostic reasoning, generative AI may assist in treatment decisions which require synthesis of complex scientific-, patient-, and systems-level factors. Generative AI solutions may be directed at all or some of these and may span levels of physician supervision.^17^ For example, new generative AI tools allow clinicians to query medical literature using free text questions and receive AI-generated answers alongside relevant citations.^18^ This is a useful capability, but the physician retains full oversight of the care process. In contrast, a large benefit of generative AI technologies is automated decision-making, and new industry entrants are currently working toward this purpose.^19–21^ Although protocol-driven care for common internal medicine activities like chronic disease management can be more effective than standard of care,^22^ automating clinical decision-making has far-reaching implications and will require rigorous evaluation standards that have not yet been implemented.

Even in its current form, this technology offers a new form of clinical decision support (CDS), with vast and generalizable medical knowledge and the ability to perform a variety of complex cognitive tasks.^23^ This may lay the foundation for more sophisticated and potentially autonomous tools for diagnosis and treatment.

### Key Challenges to Overcome

Clinical decision-making is a high-stakes activity, and generative AI currently has serious weaknesses. The most pressing challenge is its propensity to produce inaccurate information, popularly described as “hallucinations,” but more accurately described as “confabulations.”^24^ Generative AI can also fail to include important information, an error termed “omissions.” Even small errors in generative AI performance can erode physician confidence, inevitably cause patient harm, and hinder adoption.

The issue of generative AI errors and omissions is particularly salient because there appears to be a rising expectation among technologists and others that physicians will simply “supervise” generative AI tools, carefully fact-checking AI outputs for inaccuracies and mitigating discrepancies. This is a bold supposition, and we believe a frameshift among AI technologists is needed.^25^ It should not be a foregone conclusion that physicians will recognize when AI tools under-perform, nor that we will divest ourselves of our current professional practice and adopt the role of “AI supervisor.” Instead, technologists should aim to design high-performing tools that engender trust with physicians. Just like airline pilots should not need to question the accuracy of their GPS when making a flight plan, physicians should not need to question the accuracy of generative AI when designing a care plan.

### Recommendations for Enhancing Clinical Decision-Making with Generative AI

For clinicians:

- Remain attentive to developments in generative AI as a potentially transformative technology in healthcare and be receptive to using these tools in patient care.
- As with any new technology, test, or treatment, clinicians should critically appraise the value of generative AI in augmenting their practice and adopt tools that improve care.
- Recognize that errors and omissions are the major technical weakness of generative AI and understand performance and safeguards of any new tool in this domain.
- Welcome opportunities to collaborate with technologists in designing generative AI tools to improve performance and acceptability.

For technologists:

- Consider perspectives of the clinical care team in the design of generative AI tools and hold yourselves, your colleagues, and your technologies to performance standards expected of the clinical workforce.
- AI tools should ideally provide outputs that can be viewed as ground truth, but must provide obvious and intuitive mechanisms for verification and error-proofing.
- Directly partner with clinicians and patients in addition to business and technology leaders to understand real-world user needs.

For healthcare organizations:

- Evaluate generative AI tools that improve diagnosis and assist in treatment selection to reduce diagnostic error and improve achievement of therapeutic goals.
- Partner with physicians to carefully understand acceptability of new generative AI-driven workflows and responsibilities.
- When evaluating clinical decision tools, focus on preventative care and chronic condition management, as these represent the bulk of contemporary internal medicine practice with large impacts on health.

## GENERATIVE AI FOR OPTIMIZING HEALTHCARE SYSTEMS

General internists see many ways that generative AI could strengthen overall health system performance. Three major areas for consideration are improvements to access, population health management, and patient safety. Access challenges arise when the capacity of a system to deliver care is exceeded by demand. In internal medicine — and especially primary care — access has become critically limited.^26–28^ Generative AI may increase capacity through several mechanisms. First, capacity can be created when manual tasks like chart review and patient triage can be automated. New capacity could also be created if generative AI increases scope of practice, especially among advanced practice providers (APPs) who now provide substantial amounts of internal medicine services.^29^ Additionally, capacity could be enhanced with chronic and preventative care partially or fully delivered by generative AI, though such capabilities remain less studied.

Population health is another area where generative AI can extend the reach of the general internist.^30^ In the current system, it is often difficult to determine where a patient is in the care journey or identify gaps in care. Although many systems invest heavily in population health efforts, generative AI and its ability to process large amounts of data may provide needed visibility into patient progress at scale, as well as greater visibility into challenges impacting specific communities.

Additionally, generative AI represents a potential step-change in patient safety. Medical errors remain a significant concern across all specialties despite decades of efforts and national attention.^31^ Generative AI tools that can anticipate and mitigate errors automatically could provide an entirely new infrastructure on which to base patient safety systems. The transformative potential of generative AI to improve safety has attracted attention of senior leadership within industry and government, including a recent report to the president on the topic.^32^

### Key Challenges to Overcome

Integrating new technologies to create systems-level change is a difficult undertaking spanning individual and organizational factors. Key challenges include mustering leadership support, designing new workflows within complex organizations, allocating resources for implementation, building new supporting infrastructure and expertise to monitor AI performance, and overcoming institutional inertia.

### Recommendations for Improving Healthcare Systems with Generative AI

For clinicians:

- Be open to evaluating and implementing generative AI tools for quality and safety applications.
- Consider how AI tools can enhance team-based delivery of care by expanding scope of practice.

For technologists:

- Prioritize development of AI tools that address the most pressing systems-level concerns: quality and safety, access, equity, and cost.

For healthcare organizations:

- Consider opportunities for both incremental and transformational systems-level change with generative AI, with resources directed toward both.
- Ensure strong internal infrastructure is in place to monitor performance of generative AI, especially in clinical use.

## IMPROVING PHYSICIAN AND PATIENT EXPERIENCE THROUGH GENERATIVE AI

The experience of giving and receiving medical care has changed dramatically in recent decades due to factors such as the widespread adoption of electronic health records (EHR),^33–35^ reorganization of the physician workforce within large healthcare entities,^35^ value-based payment,^36^ healthcare consumerism,^36^ and the rise of telehealth and asynchronous care.^37,38^ While altruistic desires and passion for scientific inquiry often motivate individuals to pursue a career in medicine, the current practice environment poses significant challenges to professional fulfillment and cultivation of meaningful patient relationships.^39–41^

Physicians spend a significant proportion of their time on EHR documentation and other administrative tasks instead of direct patient care, and these burdens are particularly high in internal medicine.^36,42^ For instance, one recent study highlighted that primary care physicians at an academic medical center received 8000–15,000 inbox messages annually and spent 36 min on the EHR per patient visit.^43^ These increasing demands prevent delivery of comprehensive, high-quality patient care: another study estimated that a typical primary care physician needs 26.7 h per day to deliver all recommended services.^44^ These burdens also contribute to physician burnout,^45^ physician exit from clinical settings,^46^ and patient dissatisfaction.^47^

Patients are similarly affected, perceiving these challenges during their care interactions. In a recent national survey,^48^ 47% of respondents felt their healthcare providers were overburdened and 64% wished healthcare providers took more time to understand them, findings which reinforce urgency in improving the patient experience in physicians-patient interactions.

Generative AI offers an opportunity to restore humanism in medicine. Early efforts directed at reducing administrative burdens and improving workflows seek to create more time for physicians to spend with their patients. Potential use cases for the application of generative AI include chart review, clinical documentation, inbox management, personalized patient instructions, and prior authorizations.^49^ Early results using generative AI for clinical documentation found AI wrote high-quality notes, reduced documentation burden, and garnered favorable physician and patient feedback.^50^ Similarly, an early pilot using generative AI for drafting replies to patient portal messages showed favorable usability and improvements in assessments of burden and burnout, although no reduction in time was observed.^51^ In addition to administrative tasks, there are numerous other ways that generative AI may be designed to improve patient experience including more empathetic communication,^52^ improved patient instructions,^53^ and timelier answers to common patient questions.^54^

However, generative AI tools — like any other technology — require intentionality. Ideally, they will be used to fundamentally reimagine clinician and patient interactions rather than simply layered on top of dysfunctional workflows in healthcare. The promise of these technologies will be best realized through creative redesign of medical practice.

### Key Challenges to Overcome

Improvements to the patient and physician experience with generative AI require that implementations of these technologies do not substitute current workflow problems with new ones. These tools should enhance rather than diminish the patient-physician relationship. Additionally, stakeholders should avoid the temptation to “backfill” new capacity created by generative AI efficiencies, instead finding balance between increased access and improvements in experience. If generative AI becomes another distraction, a new barrier between physicians and our patients, or simply a revenue lever, an opportunity to reimagine care delivery will be missed.

### Recommendations for Improving the Physician-Patient Relationship with AI

For clinicians:

- Explore ways to leverage generative AI to create more time and attention for patients while restoring personal fulfillment in clinical practice.

For technologists:

- Although efficiency is important, understand that it is not the only desirable outcome. Strong patient-physician relationships are a critical element of healthcare delivery that create tremendous value. Generative AI tools should promote rather than hinder these interactions.
- Ensure solutions are truly improving the experience of giving and receiving care rather than simply layering on new technology.
- Co-design solutions with clinicians and patients that both incrementally improve and fundamentally redesign clinical workflows.

For healthcare organizations:

- Evaluate generative AI solutions that reduce administrative burdens as these tools are presently available, have a growing evidence base, and are demonstrating tangible benefits in improving experience for physicians and patients.
- Resist the urge to substitute the human workforce with technology solutions. Remember that the practice of medicine is a fundamentally human endeavor and that experience matters.
- Avoid solutions that simply layer generative AI on top of dysfunctional or burdensome workflows as these will have a high likelihood of failure. Reimagine workflows that make the best use of new AI capabilities.

## NAVIGATING THE ETHICAL AND EQUITY LANDSCAPE OF GENERATIVE AI IN MEDICINE

Bias in generative AI is a major concern and has been the subject of significant attention as use of these tools expands.^55–57^ In general terms, bias in generative AI can be thought of as outputs that disadvantage certain populations compared to others. For example, a generative AI trained only to classify skin lesions from white individuals may offer less accurate diagnoses and recommendations for individuals with darker skin tones.^58^ Such biases, if unrecognized, can undermine generative AI acceptability, fairness, equity, and effectiveness.

The sources of bias in generative AI are multi-dimensional and can occur at all phases of the technology life cycle.^59^ First are biases in data sets on which these solutions are built. This may be caused by inequitable participation in data sets, flaws in data collection, and erroneous characterizations. If certain groups are not well represented in generative AI training data, their specific needs may not be addressed in outputs. Examples include inequitable participation among certain races and genders, sexual orientation, pregnancy status, and others. More insidiously, generative AI systems can exhibit unanticipated biases when allowed to “learn” in an unsupervised fashion, thus perpetuating existing biases in healthcare delivery and outcomes.^60^

Additionally, bias can arise in implementation^61,62^ and reflect human rather than technology biases about where, how, and for whom generative AI is utilized.^55,63^ For example, consider a care model where patients must use AI before seeing a human. Such a system may inadvertently disadvantage some groups of patients forced to access less desirable AI-driven care. Organizations must guard against such implementation-based impacts on health equity.

Another source of ethical concern is around data privacy, ownership and monetization, and transparency. Specific legal issues notwithstanding,^64,65^ there are considerable issues of fairness and individual autonomy created when personal data is used to train generative AI. This presents a dilemma: use patient medical records to train generative AI in the interest of the greater good through improved performance or undertake potentially burdensome informed consent efforts which may hamper improvements. Generative AI — and the financial incentives to monetize new tools — creates new pressures to loosen historical restrictions on use of health data, which may erode trust.

The proprietary nature of generative AI tools also poses ethical challenges around knowledge sharing and financial conflict of interest. The development of AI models is a capital-intensive endeavor dependent upon corporations with a primary profit objective. These entities may not be incentivized to share best practices or advancements, but instead to maintain a competitive advantage through proprietary technology, market domination, and curated evaluations of performance that demonstrate success, not failure. While these strategies are common in industry, these values can conflict with the primary objective of healthcare stakeholders seeking to improve health outcomes. In the pharmaceutical industry, this tension is mitigated by requiring manufacturers to produce extensive safety and efficacy studies as pre-requisites to regulatory approval followed by a period of profitable market exclusivity before introduction of low-cost generics. Although the Food and Drug Administration has proposed regulatory oversight of AI, it does not yet approach the rigor of pharmaceutical regulation.^66^ Moreover, there is an ethical dilemma inherent to sequestering technological advancements within industry when broader sharing of such advancements may substantially benefit society. Users of generative AI in healthcare must also recognize the potential for underlying financial conflicts of interests influencing AI outputs, such as AI designed to optimize healthcare revenue rather than optimize health outcomes.

A lack of transparency regarding the design and performance of generative AI tools, as well as the “black box” nature of AI decision-making processes, also introduces tremendous uncertainty for those adopting these solutions and makes it difficult for individual clinicians to act on AI-generated recommendations. Clinicians and patients need clear evidence of AI performance coupled with understandable ways to interpret generative AI outputs beyond “the AI said so.” Both existing and new techniques will likely be required. For example, a “chain of thought” approach can provide a step-by-step breakdown of the reasoning process behind a particular recommendation. Existing AI-based tools like the Epic Deterioration Index^67^ already include features which allow physicians to understand specific contributing factors behind AI-based recommendations. Additional techniques like visualizations and natural language explanations can further enhance transparency, making AI recommendations more trustworthy and understandable.

Finally, generative AI tools raise important questions of scope. Medicine is best understood not only as a science but as a “moral practice” requiring human to human interactions.^68^ If generative AI algorithms come to define the standard of care, they may undermine physicians’ ability to connect with patients and exercise clinical discretion. For example, an insurer might require that generative AI evaluates a patient and only reimburses orders that the AI deems necessary, inappropriately narrowing the scope of physician autonomy. Competing AI tools designed for different purposes — for example, a clinical recommendation versus prior authorization approval — could also yield conflicting recommendations. These concerns call for physicians to define the appropriate use of generative AI involvement in decision-making before these tools arrive at the bedside, and to clearly articulate the value of human judgment.

### Key Challenges to Overcome

Financial incentives are already placing immense pressure on technology organizations to bring generative AI tools to markets, and the value set of industry fundamentally differs from the values of the medical profession. This distinction may manifest in accelerating tools to market despite inadequate assessment and mitigation of bias, through undesirable tactics to maintain market domination at the expense of patient care, and diminution of the human aspects of care delivery.

### Recommendations for Navigating Ethical and Equity Issues in Generative AI

For clinicians:

- Insist on high standards of transparency and evidence for AI tools — including AI’s potential for bias (differential performance).
- Do not use generative AI tools to make clinical decisions unless confident that you can justify those decisions to patients and peers.

For technologists:

- Seek to address bias in generative AI performance through more representative training data, evaluation and mitigation of bias in outputs, and ongoing monitoring of performance.
- Fund high-quality studies of generative AI performance in the form of both clinical trials and real-world outcomes evaluations.
- Recognize that ethical standards differ between healthcare and business organizations and create internal systems of checks and balances to navigate tensions similar to other high-stakes engineering domains such as aerospace, nuclear energy, and automotive safety.
- Approach the design of AI tools with the mindset that they should work to augment clinicians rather than clinicians augmenting generative AI.
- Seek to understand the perspectives of patients and community organizations when assessing the equity impact of generative as these stakeholders are often able to surface important concerns early in the adoption process.

For healthcare organizations:

- Demand diverse training data sets, transparency into performance, and equitable outcomes in order to promote fairness when using generative AI.
- Ensure physicians maintain agency and ultimate decision-making authority, irrespective of generative AI recommendations.
- Critically evaluate data on generative AI performance when making adoption decisions, recognizing that industry may have different incentives than healthcare organizations.

## CONCLUSION

Generative AI will undoubtedly impact healthcare in ways both predictable and unpredictable, and there is tremendous promise for positive impact on care delivery, clinician and patient experience, equity, and cost of care. However, choices made in the near-term may have far-reaching consequences for the medical profession broadly and general internal medicine in particular. Embedded in these recommendations are key themes that can guide decisions across stakeholders: a focus on deploying this new technology to enhance rather than impede care, the need for rigorous evaluation and supporting institutional structures to guide generative AI development and implementation, and the recognition that the practice of medicine is, and must remain, a deeply human endeavor. This position statement serves as an important guidepost for all those exploring how generative AI tools may benefit medical practice while guarding against the potential pitfalls of implementing this new technology at scale.

## Supplementary Information

Below is the link to the electronic supplementary material.

Supplementary file1 (DOCX 1140 KB)
