# Supplementary material for: Recommendations for Clinicians, Technologists, and Healthcare Organizations on the Use of Generative Artificial Intelligence in Medicine: A Position Statement from the Society of General Internal Medicine
Source: J Gen Intern Med. 2024 Nov 12;40(3):694–702. doi: 10.1007/s11606-024-09102-0 (PMC11861482; doi:10.1007/s11606-024-09102-0)

### Supplementary Appendix 1: Examples of Generative AI Capabilities

1. *Natural Language Prompting and Responses:* Generative AI tools can understand questions posed in natural language and then generate accurate responses to those questions. In this example, the LLM (GPT-4, OpenAI) is asked to summarize an HPI into the most important findings and produce a most likely diagnosis.


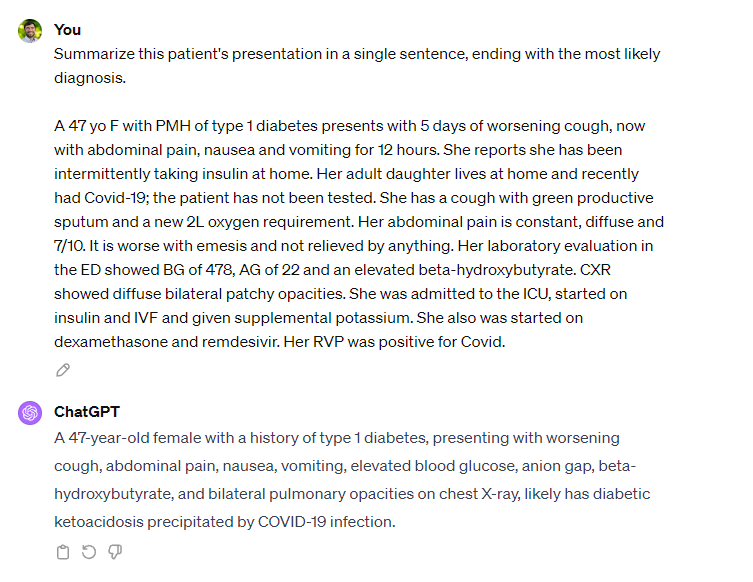


1. *Demonstration of Reasoning*: When asked to further explain its reasoning, GPT-4 is able to articulate a rationale for selecting DKA provoked by Covid-19 as the correct diagnosis, even though DKA is not mentioned in the HPI. Note that GPT-4 has never been trained on this specific question, but is able to leverage its extensive training data to ‘know’ that DKA can be provoked by infections, that this patient’s labs and symptoms suggest DKA, and that the presence of concurrent severe Covid-19 infection strongly suggests this as the precipitant. Also note how it is able to explain itself in clear and understandable natural language.


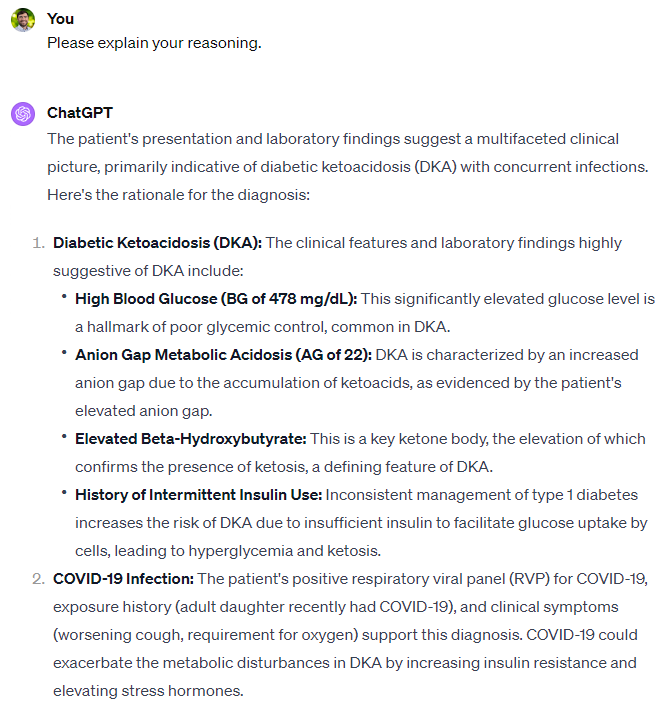


C) *Creating useful content*: The ‘generative’ in generative AI refers to its ability to actually create content. Below, GPT-4 creates a description of DKA appropriate for a lay audience to help educate the imaginary patient’s family. However, note that GPT-4 is not simply parroting off pre-determined facts about DKA, but weaving in the patient’s known context and other diagnoses. GPT-4 is also able to understand that speaking with a patient’s family requires simplification of information and empathetic tone, which it further integrates.


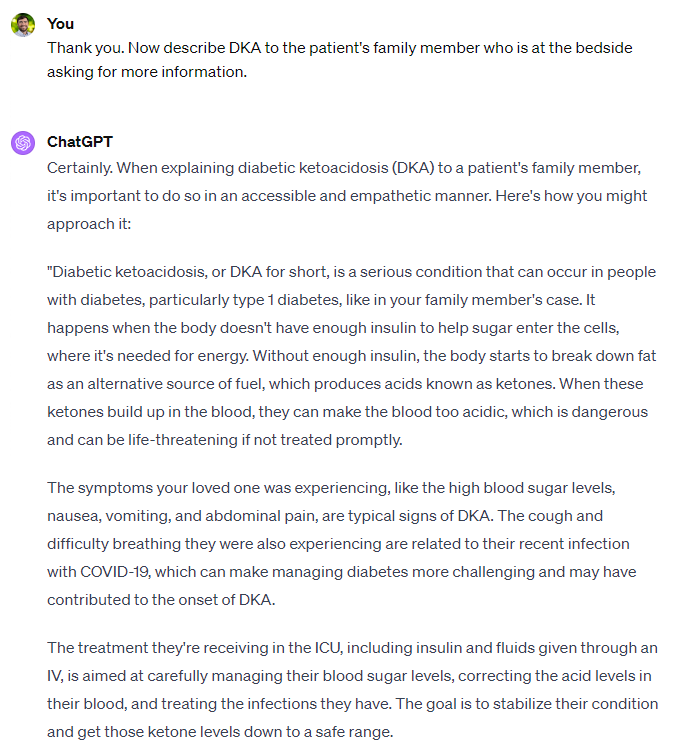


D) *Categorization and Synthesis of Key Information:* LLMs like GPT-4 distill key information from complex text. In this example, GPT-4 identifies the specific medical problems from the patient’s admission to produce a short problem list for insertion into the discharge summary.


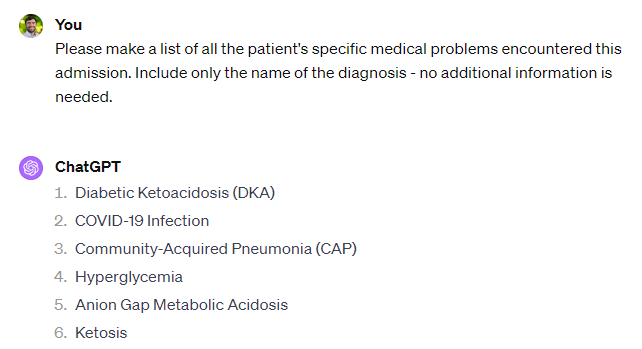


E) *Natural language search*: Generative AI models are able to leverage their natural language capabilities to understand the content in text-based documents, and then retrieve relevant search results. OpenEvidence is one such tool allowing clinicians to use free-text search to identify and retrieve relevant sources to support medical decision making. In this example, the generative AI is asked about current HFrEF guidelines and quickly returns an answer referencing the specific passages from the ACC guidelines supporting the AI’s answer.


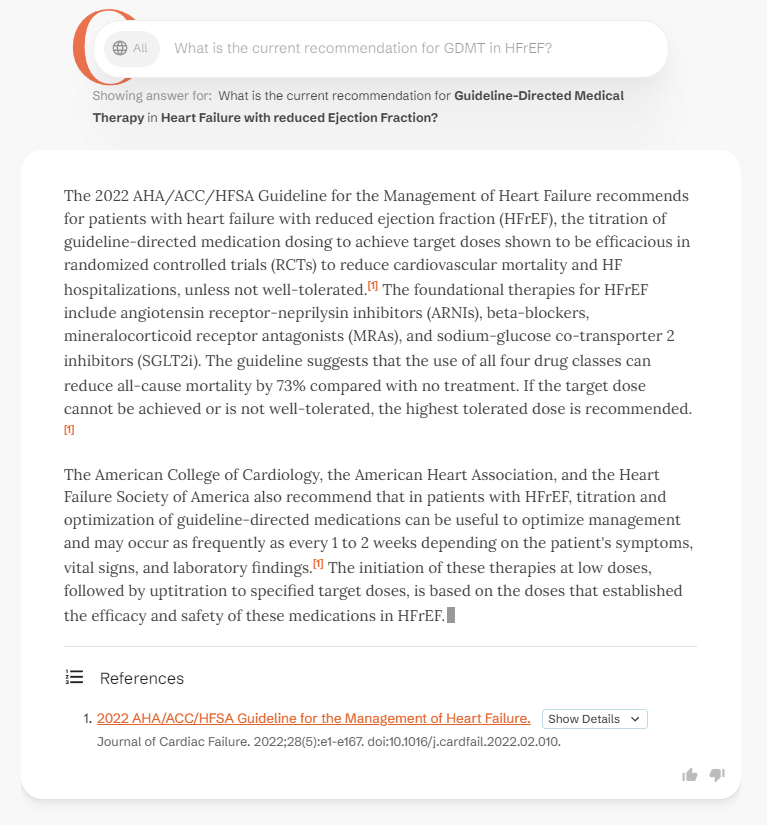


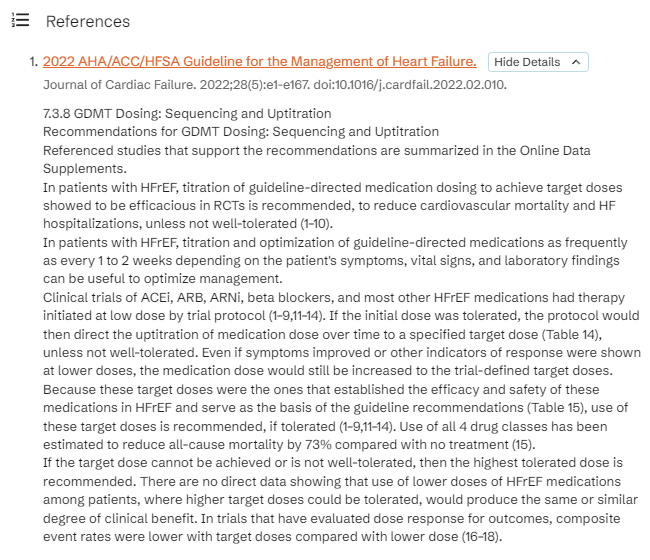


F) *Speech-to-Text and Audio Capabilities*: Generative AI tools are able to process human speech and transform it into text. This capability extends beyond simple transcription; these tools can analyze the context and details of the speech and transform dialogue into important components. This capability is currently in use as “ambient scribing” in which a generative AI ‘listens’ to a patient/physician interaction and creates structured documents such as an HPI or even an entire clinical note. Similarly, text can be converted into audio, including the ability to create speech in a variety of voice types.

G) *Image Generation and Interpretation:* LLMs are able to generate images from natural language by training the AI to associate images with words. In the examples below, GPT-4 produces an image of doctors and nurses, although it had to be prompted again to produce a more diverse image reflecting the current healthcare workforce (example of bias). In the second example, GPT-4 is handed an image and is able to correctly describe the scene.


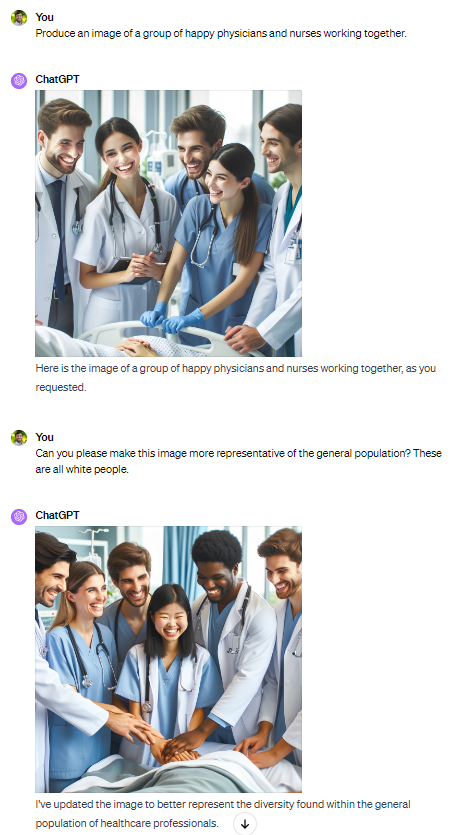


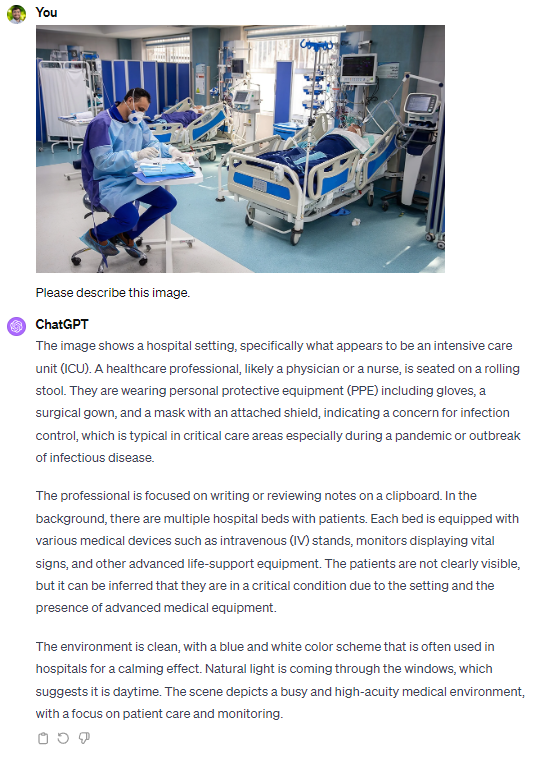

Supplement: Supplementary file 1 — Supplementary file1 (DOCX 1140 KB) [file 11606_2024_9102_MOESM1_ESM.docx]
